# Perfusion System for Modification of Luminal Contents of Human Intestinal Organoids and Realtime Imaging Analysis of Microbial Populations

**DOI:** 10.3390/mi13010131

**Published:** 2022-01-14

**Authors:** Nicholas J. Ginga, Raleigh Slyman, Ge-Ah Kim, Eric Parigoris, Sha Huang, Veda K. Yadagiri, Vincent B. Young, Jason R. Spence, Shuichi Takayama

**Affiliations:** 1 Wallace H. Coulter Department of Biomedical Engineering, Georgia Institute of Technology and Emory School of Medicine, Atlanta, GA 30332, USA; nick.ginga@gatech.edu (N.J.G.); rslyman3@gatech.edu (R.S.); geahkim@gatech.edu (G.-A.K.); eparigoris3@gatech.edu (E.P.); 2 The Parker H. Petit Institute of Bioengineering and Bioscience, Georgia Institute of Technology, Atlanta, GA 30332, USA; 3 School of Materials Science and Engineering, Georgia Institute of Technology, Atlanta, GA 30332, USA; 4 Division of Gastroenterology, Department of Internal Medicine, University of Michigan Medical School, Ann Arbor, MI 48109, USA; shahuang@umich.edu (S.H.); spencejr@umich.edu (J.R.S.); 5 Division of Infectious Diseases, Department of Internal Medicine, University of Michigan Medical School, Ann Arbor, MI 48109, USA; vyadagir@med.umich.edu (V.K.Y.); youngvi@umich.edu (V.B.Y.); 6 Department of Microbiology and Immunology, University of Michigan Medical School, Ann Arbor, MI 48109, USA; 7 Department of Cell and Developmental Biology, University of Michigan Medical School, Ann Arbor, MI 48109, USA; 8 Department of Biomedical Engineering, University of Michigan Medical School, Ann Arbor, MI 48109, USA

**Keywords:** perfusion, organoid, bacteria, intestine, microfluidics, pump, injection, PDMS, image analysis, fluorescence

## Abstract

Intestinal organoids are 3D cell structures that replicate some aspects of organ function and are organized with a polarized epithelium facing a central lumen. To enable more applications, new technologies are needed to access the luminal cavity and apical cell surface of organoids. We developed a perfusion system utilizing a double-barrel glass capillary with a pressure-based pump to access and modify the luminal contents of a human intestinal organoid for extended periods of time while applying cyclic cellular strain. Cyclic injection and withdrawal of fluorescent FITC-Dextran coupled with real-time measurement of fluorescence intensity showed discrete changes of intensity correlating with perfusion cycles. The perfusion system was also used to modify the lumen of organoids injected with GFP-expressing *E. coli*. Due to the low concentration and fluorescence of the *E. coli*, a novel imaging analysis method utilizing bacteria enumeration and image flattening was developed to monitor *E. coli* within the organoid. Collectively, this work shows that a double-barrel perfusion system provides constant luminal access and allows regulation of luminal contents and luminal mixing.

## 1. Introduction

Organoids are 3D cell cultures that arise from the self-organization of adult or pluripotent stem cells into organotypic structures [1,2,3]. Organoids often have fluid-filled lumens that are inaccessible from the outside, making direct interaction with the apical surface difficult [4]. Accessing the apical surface is required for some studies, such as host–microbe interactions which take place on the luminal surface of the epithelium. For such studies, growth of apical-out enteroids has been developed, but other methods are still needed [4,5]. One such method to access the apical cell surface is to create 2D cell cultures from 3D organoids. Although this enables the use of established 2D cell culture test methods, it adds additional steps and processes to create 2D cell cultures from 3D organoids [6,7,8]. An approach that utilizes organoids in their 3D cell structure and allows access to the lumen and apical cell surface of the closed organoid structure is to perfuse the interior of organoids by glass capillary microinjection. This is a straightforward approach to regulate the luminal environment and control the luminal volume [9,10]. Technologies that interface with the luminal environment can improve the physiological accuracy of organoid models consisting of a wide range of tissues, such as intestine [11,12,13], lung [14,15], or cancer [16,17], where interactions with the apical surface are important. Specifically, in pluripotent stem-cell-derived human intestinal organoids (HIOs), interactions between bacteria injected into the lumen and the apical surface have been shown to induce phenotypic changes that cause HIOs to resemble more mature tissue [18]. In such situations, continuous access to the luminal contents and regulation of the contents with the use of a glass capillary perfusion system offers advantages to improve the physiological relevance of HIOs.

The work presented herein demonstrates the potential of perfusion systems to regulate a luminal microbiome within an HIO. In adapting approaches from prior organoid perfusion methods [9,10], design changes were made to increase the success rate of perfusion and allow for control of luminal mixing. This was achieved by using a double-barrel capillary and pressure-based pump in the perfusion system. A double-barrel capillary requires only one puncture of an HIO to provide separate flow channels for injection and withdrawal of substances to and from the lumen. In comparison, the use of two single-barrel capillaries requires two alignments and punctures of the capillaries with the HIO, which can be time-consuming and lead to lower success rates. Additionally, pressure-based pumps provide improved temporal control of flow, compared with displacement-based pumps, through small-diameter passages such as those found in pulled glass capillaries. Moreover, to image fluorescent bacteria within the lumen of an HIO spanning across a wide focal range, an imaging approach was developed that utilizes bacteria enumeration instead of fluorescence intensity. The results show that the perfusion device was able to modulate luminal contents and bacterial proliferation. Additionally, the device applied periodic strain to the epithelium of the organoids while maintaining a relatively constant average volume over the course of the experiment. The histology of HIOs colonized with pathogenic *E. coli* and perfused during bacteria growth demonstrates a clear cellular response to perfusion, providing a proof-of-concept for the current perfusion and imaging methodology.

## 2. Materials and Methods

### 2.1. Perfusion System Fabrication

#### 2.1.1. HIO Holder

**Figure 1:**
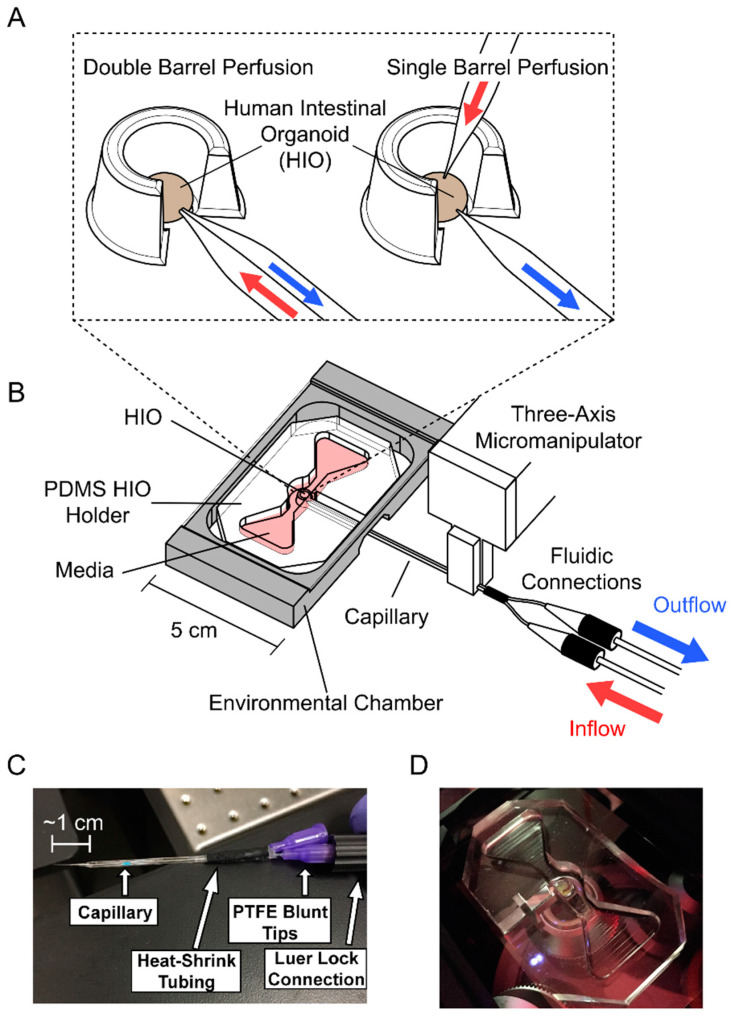
(**A**) A zoomed-in schematic of the human intestinal organoid (HIO) puncture strategy with one double-barreled capillary or two single-barreled capillaries. (**B**) The device utilizes a micromanipulator to position the capillary that punctures the HIO. The HIO is placed in a PDMS HIO holder to assist with the puncture and maintain environmental conditions. (**C**) A photo of a double-barreled capillary shows the components that allow for an air-tight connection with the fluidic lines. (**D**) A photo of an HIO and Matrigel within the PDMS HIO holder without media.

The main function of the HIO holder is to immobilize the HIO while also providing access to the HIO for a horizontally positioned double-barrel glass capillary controlled by a micromanipulator to pierce and then inject/withdraw material from the HIO (Figure 1). The HIO is immobilized by placing it in a semicircular well defined by poly(dimethyl siloxane) (PDMS, Sylgard 184, Dow Corning, Midland, MI, USA) walls with tapered widths, as shown in Figure 1A. The tapered walls allow a range of HIO diameters to be held securely within the HIO holder. The HIO holder also contains a reservoir of growth media surrounding the HIO to keep it immersed for extended periods. The HIO holder is designed to allow brightfield and fluorescence imaging using an inverted microscope during the entire setup and perfusion process. The mold for the HIO holder (Figure 1B) was 3D printed by Protolabs, Inc. with Watershed XC 11122 resin. The mold was filled with PDMS at a 10:1 (*w*/*w*) base polymer to curing agent ratio. PDMS was poured into each mold at the minimum amount required to cover and submerge the mold’s features. This was to create a PDMS thickness of the bottom of each HIO holder thin enough (~500 µm) to allow the working distance (14.0 mm for 5× and 11.1 mm for 10×) of the microscope to image the entire height of the HIO while in the holder. The HIO holder was set at 65 °C overnight before being removed from the mold.

#### 2.1.2. Perfusion System Assembly

The PDMS HIO holder was placed on top of a glass cover slide, which was then placed into a larger 3D-printed platform. This platform had a micromanipulator (DT12XYZ, ThorLabs, Newton, NJ, USA) attached to it and was used for the positioning of the glass capillary in the x, y, and z directions. A 3D-printed fixture to hold a double-barrel glass capillary was attached to the micromanipulator (Figure 1B). The 3D-printed platform of the system provided a stationary base to maintain the relative position between the HIO holder and the double-barrel capillary attached to the micromanipulator. This facilitated the process of aligning the tip of the double-barrel capillary with the HIO and the subsequent piercing of the HIO to gain luminal access of the HIO.

#### 2.1.3. Capillary Tip Fabrication

Previous organoid perfusion systems predominantly utilized two single-barreled glass capillaries to inject and withdraw fluid [9,10]. The presented design instead uses a single double-barreled glass capillary, with each barrel acting as a separate flow channel. Use of a double-barreled capillary requires only a single puncture of the organoid and thus increases the likelihood of a successful experiment. The presented system can be adapted for two single-barreled glass capillaries if a second micromanipulator is used above the device (Figure 1A).

Double-barreled borosilicate glass capillaries with an outer diameter of 1.0 mm and inner diameter of 0.75 mm and single-barrel borosilicate glass capillaries with an outer diameter of 1.5 mm and inner diameter of 0.86 mm were acquired from Sutter Instruments Company. Capillary sizes were selected to optimize tip geometry while maintaining compatibility with existing microfluidic connectors. Capillaries were pulled using a PC-10 Puller (Narishige Group, Amityville, NY, USA) to achieve a longer pointed tip that tapered from its full diameter to its tip over a length of ~1 cm (Figure 1C) (details in Supplementary Information Section S1). In comparison to the more commonly produced short “bee-stinger” style tips, a longer and narrower tip could access the center of the lumen without causing significant stretching of the HIO puncture site.

The double-barreled capillary tip diameter was measured as the maximal length across the end of the sharpened tip. Tip sizes between 100 μm and 120 μm were used interchangeably for various perfusion experiments, but a 100 μm diameter was targeted, as smaller tip sizes could result in clogging of the withdrawal barrel during operation, and larger tip sizes made puncture of the HIO difficult. For the fluorescein isothiocyanate-dextran (FITC-dextran) perfusion, the double-barreled capillary had a 120 μm diameter. For the *E. coli* perfusion, the double-barreled capillary had a 105 μm diameter and the single-barrel glass capillary used for bacterial injection had a 10 μm diameter. Injection capillaries were less prone to clogging, so smaller tip sizes could be used.

To allow for Luer lock connections, polytetrafluoroethylene blunt-tip pipettes (0.24 mm outer diameter, 0.12 mm inner diameter, Jensen Global Inc., Santa Barbara, CA, USA) were inserted into both barrels of the double-barreled glass capillaries. To prevent leakage, the interface between blunt-tip pipette and glass capillaries was sealed with silicone adhesive. Heat shrink tubing was placed around the silicone and shrunk to ensure a complete seal (Figure 1C). The silicone was allowed 24 h to set before the capillaries were used. With the blunt-tip pipettes securely attached to each of the barrels of the double-barrel capillary, they could then be connected easily to the tubing of the Elveflow pump (OB1 MKIII+, Elveflow, Paris, France) (Figure S1 in Supplementary Information). The Elveflow pump is a pressure-based pump designed specifically for microfluidic systems. Such pressure-based pumps allow precise control of small fluid volume delivery and greater temporal control of fluid flow, which is desirable for implementation of cyclic flow compared with displacement-based pumps. The flow rate of the pulled glass capillary utilized during perfusion is a function of the applied pressure from the pump. Measurements to investigate the relationship between the flow rate and applied pressure are presented in the Supplementary Information (Figure S2).

### 2.2. HIO Production

Pluripotent stem-cell-derived HIOs were generated following the procedure defined in McCracken et al. [3] and Hill et al. [19]. The complete protocol for generation and maintenance of HIOs can be found in Sections 4 and 5 of Hill et al. [19]. HIOs were ~50 days old on the day of perfusion for both experiments. Control and test HIOs used in the bacterial perfusion experiment were from the same initial batch. HIOs were embedded in a growth-factor-reduced Matrigel dome (Cat# 356231, Corning Inc., Corning, NY, USA) with a protein concentration of >8 mg/mL to replicate physiological extracellular matrix support and stiffness. Then, 500–750 µL of HIO maintenance media [3] was added to each well and replaced every 2–3 days.

### 2.3. Perfusing HIOs with FITC Dextran

HIOs were extracted from the Matrigel using a pipette tip and a single HIO was pipetted into the central chamber of each HIO holder. Matrigel was dispensed on top of the HIO to further limit movement. HIO media was dispensed into the holder’s reservoir to cover the HIO. Both barrels of a double-barreled glass capillary were filled and primed with phosphate-buffered saline (PBS) by applying a constant positive pressure to both barrels.

A negative pressure of 200 mbar was applied for 45 s to draw up FITC-Dextran 4k (TdB Labs, Uppsala, Sweden) into the injection barrel of the double-barreled glass capillary. The micromanipulator was utilized to position the capillary and puncture the HIO with the capillary tip. The capillary was further inserted such that the capillary tip resided in the center of the HIO lumen.

No flow was applied for 5 min to allow the HIO to seal around the capillary. If sealing did not occur, this was signified by an observation of a decrease in the diameter of the HIO. Five minutes after puncture, the HIO was subjected to perfusion cycles. Each perfusion cycle consisted of 3 s of injection, a 5 s pause, and then 3 s of withdrawal. After 20 perfusion cycles were performed, the HIO had expanded, so withdrawal was performed to return the HIO to its original size. Lastly, constant injection and withdrawal were simultaneously performed for an extended duration to wash out the HIO. This perfusion experiment was conducted with one HIO. Detailed information on the pressures and flow sequence can be found in the Supplementary Information (Section S3).

### 2.4. Colonizing HIOs with GFP-Labeled E. coli and Perfusion

Enterohemorrhagic *E. coli* (EHEC) 0157:H7 labeled with green fluorescent protein (GFP) (ATCC 51657GFP) were cultured in tryptic soy broth supplemented with 1% glucose until the mid-exponential growth phase and subsequently diluted to reach a concentration of 2.2 × 10^8^ colony-forming units (CFU)/mL. The bacteria were centrifuged at 4000× *g* for 5 min, resuspended in PBS, diluted to reach 1 × 10^7^ CFU/mL, and vortexed.

A single-barrel glass capillary with a 10 μm tip diameter was connected to a MINJ-D microinjector (Tritech Research, Los Angeles, CA, USA). Negative pressure was applied to the capillary to draw up an excess of *E. coli* suspension. Using an MMO-220C micromanipulator (Narishige Group, Amityville, NY, USA), the capillary punctured and injected HIOs with the same flow parameters (details included in Supplementary Information Section S4). This resulted in an estimated *E. coli* injection of 650 CFU/HIO.

The HIOs were allowed to rest for 5 min for the capillary puncture hole to seal, after which they were submerged in HIO growth media with 1× Penicillin-Streptomycin (P/S) (Thermo Fisher Scientific, Waltham, MA, USA) and incubated at 37 °C and 5% CO_2_ for 30 min. P/S was used to prevent growth of any bacteria external to the HIO that may have resulted from the bacterial injection process.

**Figure 2:**
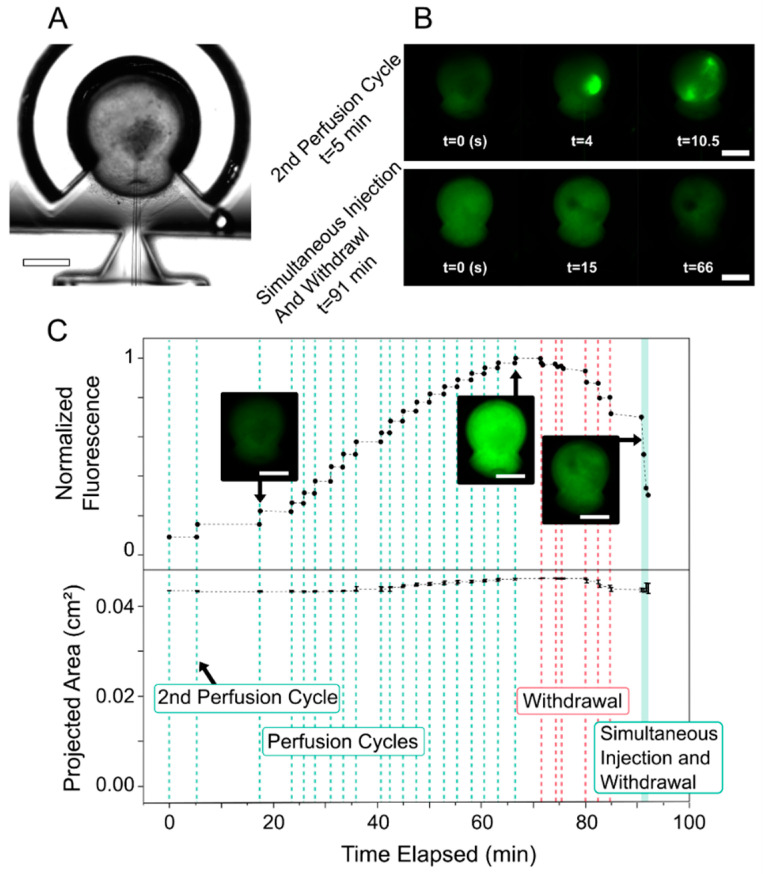
(**A**) Brightfield image of a punctured HIO in a PDMS holder. Scale bar: 1 mm. (**B**) Towards demonstrating convective mixing, selected fluorescence images are shown from near the beginning (t = 5 min) and end (t = 91 min) of the experiment. The second perfusion cycle shows injection (t = 4 s) and withdrawal (t = 10.5 s), whereas the simultaneous injection and withdrawal (constant perfusion) shows more homogenous decrease in fluorescence over time. Scale bars: 1 mm. (**C**) Normalized fluorescence (with respect to the maximum value) and HIO projected area plotted with time. The mean values of three separate fluorescence measurements are plotted as dots. The standard deviation for each timepoint was <0.004, so error bars are not visible on the graph. On the lower half of the graph, ±1 standard deviation of the projected area is plotted. Applied perfusion cycles are shown with green dotted lines, perfusion cycles consisting of only withdrawal are shown with red dotted lines, and constant simultaneous injection and withdrawal is shown in the green shaded region. Note: fluorescence before the first perfusion cycle is not included. Plotted data is for one HIO.

The control HIO was placed in a four-well plate with HIO growth media (−P/S) and left at room temperature. The HIO to be perfused was placed into the PDMS HIO holder (Figure 2A). Matrigel was dispensed on top of the HIO, and the holder’s media reservoir was filled with HIO growth media (−P/S).

Both the barrels of a double-barrel glass capillary (tip diameter = 105 μm) were filled and primed with PBS and then enough growth media to supply the perfusion experiment was drawn up into the injection barrel. Then the double-barrel glass capillary was placed into the micromanipulator. Using the micromanipulator, the capillary punctured the membrane of the HIO and was maneuvered to the center of the lumen. After allowing the HIO 5 min to form a closure around the capillary puncture site, a periodic perfusion sequence was performed for 6.5 h. One iteration of the perfusion sequence consisted of three consecutive cycles of injection and withdrawal followed by a 30-min pause. Detailed information on the pressures and flow sequence can be found in the Supplementary Information (Section S4). Optical and fluorescence images were taken every 5 min. After the perfusion had finished, the HIO had decreased in size and was expanded to its initial size for final imaging. Two perfusion experiments were conducted, each consisting of one perfused HIO colonized with *E. coli* and another HIO colonized with *E. coli* and not perfused. The results of one of these sets of experiments are presented and discussed in Section 3.2, Section 3.3 and Section 3.5.

### 2.5. Image Analysis

All image analysis was conducted using Fiji (National Institutes of Health) [20] and additional information on the image analysis scripts is provided in the Supplementary Information (Sections S5 and S6). For the dye perfusion, brightfield images were used to manually define a region of interest around the HIO. The mean pixel value of the corresponding fluorescence image within this region was recorded. Each measurement was performed three times to account for human error in defining the region of interest.

For the *E. coli* perfusion experiment, the fluorescence from the *E. coli* was of comparable intensity to the autofluorescence of the surrounding HIO media. Therefore, bacteria were instead counted using the ‘Find Maxima’ algorithm in Fiji with a prominence of 500. The peaks were limited to a region of interest around the HIO, which was manually defined using corresponding brightfield images. Cell enumeration algorithms have been used for high magnification image analysis [21]; the method utilized herein has been adapted for low magnification and does not involve image segmentation.

**Figure 3:**
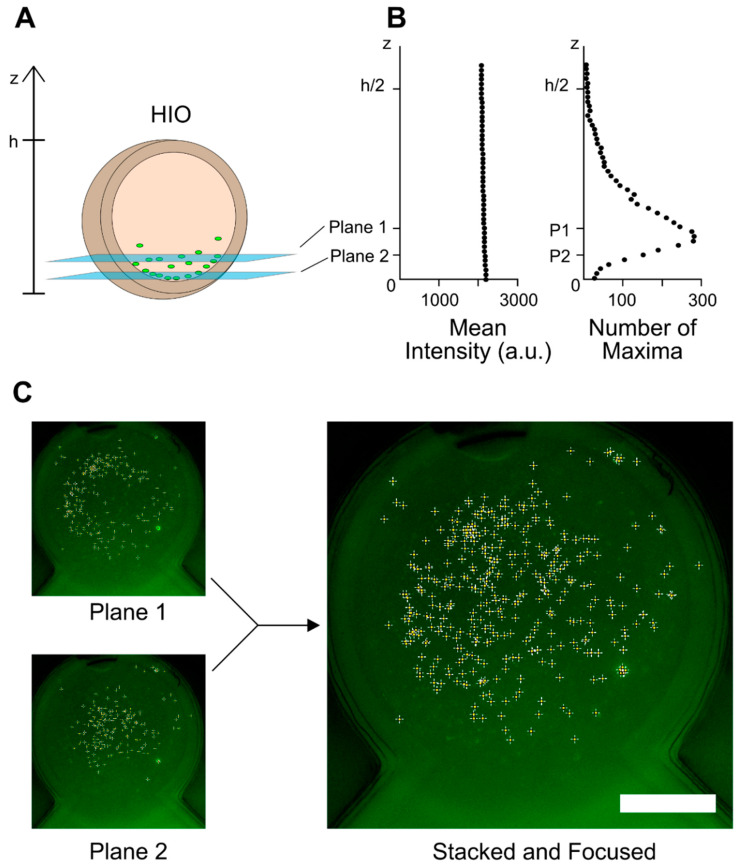
(**A**) The E. coli primarily resided on the lower luminal wall, so focal planes were selected to capture a maximal number of distinct bacteria. The plotted data is for one HIO. (**B**) Within the circular region defined by the HIO, mean fluorescence intensity and number of identified maxima are shown as a function of height z, with z = 0 corresponding to the bottom of the HIO. (**C**) Separate planes are flattened into a single plane for subsequent image analysis. Identified bacteria are marked with plus signs. Scale bar: 500 μm.

For initial and final measurements that included multiple planes, individual images were flattened into a single, focused image. This was accomplished by first adjusting the histograms of the images to have the same brightness. Then, a stack-focusing plugin was used. This software uses edge detection to identify in-focus parts of the images and combine them into a single, focused image (Figure 3C). The resulting image, which is referred to as the flattened image, has an effectively wider depth of field than would be possible in a single capture. This procedure was adapted and simplified from established methods for extending depth of field with multiple focal planes [22].

### 2.6. Organoid Histology

Organoid histology was performed based on our previously described protocol [17]. Briefly, organoids were harvested and fixed in 4% paraformaldehyde (Alfa Aesar, Haverhill, MA, USA) for 1 h at room temperature. Organoids were incubated in a 30% (*w*/*v*) sucrose solution at 4 °C for 48–72 h, or until organoids sunk to the bottom of the tube. Organoids were stained with 0.5% methylene blue for 10 min at room temperature and placed in optimal cutting temperature (OCT) compound (Tissue-Tek^®^, Sakura, Torrance, CA, USA) in a cryomold. Isopentane was cooled in liquid nitrogen, and organoids were then flash frozen. A Cryostar NX70 cryostat (Thermo Fisher Scientific, Waltham, MA, USA) was used to obtain 10 μm sections.

For immunofluorescence staining, sections were thawed to room temperature, washed with PBS, and permeabilized with 0.2% Triton X-100 (Sigma-Aldrich, St. Louis, MO, USA) for 5 min at room temperature. After PBS rinses, sections were blocked with 4% BSA in PBS for one hour at room temperature. The antibodies used in this work are listed in Table S1 in the Supplementary Information. Primary antibodies were incubated with the samples in 1% bovine serum albumin (BSA) in PBS overnight at 4 °C. After removing the primary antibody solutions, slides were rinsed three times in 1% BSA in PBS. Secondary antibodies were added to the slides and incubated for 2 h at room temperature. Finally, DAPI was added to the slides for 15 min at room temperature. Slides were then mounted with mounting media and coverslipped. Mounted slides were imaged on a DMi8 epifluorescence microscope (Leica Microsystems Inc., Buffalo Grove, IL, USA) using HC PL APO 20x/0.80 DRY and HC PL APO 63x/1.40 OIL objectives. For H&E staining, thawed sections were stained with an ST5010 Autostainer XL (Leica Biosystems Inc., Buffalo Grove, IL, USA). After coverslipping samples with Xylene and Cytoseal 60, slides were imaged with a DMi1 microscope with a color camera and 5× and 10× objective.

## 3. Results and Discussion

Two main experiments were performed to determine the perfusion system’s ability to regulate luminal contents of an HIO and are discussed subsequently. In the first experiment, the intensity of fluorescent dye within an HIO was measured as the perfusion system cyclically injected fluorescent dye into the lumen and then withdrew luminal contents. A second experiment utilized the system to perfuse a culture of pathogenic *E. coli* within the HIO’s lumen. Differences in experimental and analytical approaches between the two experiments underscore key design considerations in perfusing and imaging a large 3D environment and are discussed in the following sections.

### 3.1. Fluorescent Dye Perfusion Experiment

The perfusion system’s ability to transfer fluid within the lumen was assessed through a fluorescent dye (FITC-dextran) perfusion experiment. Intensity measurements (Figure 2C) displayed discrete jumps in fluorescence corresponding with each perfusion cycle. As the projected area of the HIO only varied by 6% from its initial value at its peak, changes in fluorescence are attributable mostly to the addition and removal of luminal dye and less to size changes.

The initial increase, leveling off, and decrease of fluorescence can be explained by the finite amount of dye loaded into the injection barrel. The injection barrel was loaded with dye for 45 s, corresponding to ~15 injection cycles. Although all perfusion cycles consisted of the same amount of injection and withdrawal, initial cycles resulted in the largest increases in fluorescence whereas later cycles showed diminished fluorescence gain. This decrease in fluorescence gain per cycle leading up to cycle 20 may have been caused by diffusion of the dye into the PBS working fluid within the glass capillary before injection. This caused the concentration of dye being injected to be reduced for the later injections. Moreover, simultaneous injection and withdrawal at the end of the experiment reduced fluorescence intensity by essentially performing a “wash-out”. These observations suggest that new fluid can be introduced to the luminal space and subsequently washed out with further perfusion.

Fluidic transfer also introduces convective mixing, which is integral for the perfusion device to affect the entire luminal volume’s contents. Such mixing was observed after dye was injected into a volume adjacent to the capillary tip, and the withdrawal process caused mixing of the luminal environment and dispersion of the dye into the entire luminal space (Figure 2B). After the first few perfusion cycles, enough convective mixing had occurred such that the fluorescence was distributed evenly throughout the HIO (Figure 2C, leftmost inset image). Convective mixing during the simultaneous injection and withdrawal at the end of the experiment was substantial enough to result in a homogenous decrease in fluorescence within the lumen and not just around the capillary tip (Figure 2B, Supplementary Video S1). Accordingly, sufficient convective luminal mixing is an important consideration in regulating the entire luminal space.

The role of timed convective mixing in regulating luminal contents highlights the benefit of using a pressure-based pump. Pressure-based pumps modulate flow at the capillary tip, which can be initiated and stopped almost instantaneously. Conversely, when the use of displacement-based pumps, such as syringe pumps, was attempted in these experiments, this resulted in flow rates which gradually decreased over time, long after the syringe had been depressed. This effect is likely caused by the high fluidic resistance of the tip, which causes pressure buildup in the syringe and tubing due to capacitance that is gradually equilibrated. For applications where temporal considerations of flow and mixing are important (e.g., cyclic operations as opposed to constant, long-term, single operation), pressure-based pumps are less affected by the hydraulic resistance and capacitance of the system and therefore afford greater temporal control. The temporal control of the fluid flow is extremely important due to the cyclic nature of the injection and withdrawal processes of the perfusion occurring over extended periods of time and is why the pressure-based Elveflow pump was implemented in the perfusion system.

### 3.2. GFP-Labeled E. coli Imaging

When adapting the perfusion system to regulate GFP-fluorescent *E. coli*, imaging and image analysis required an approach different from dye imaging. As the GFP-labeled *E. coli* had less fluorescence intensity than the FITC-dextran dye, autofluorescence of the media and HIO now contributed significantly to the mean fluorescence intensity. Thus, the mean fluorescence intensity showed no obvious correlation to visible bacteria concentration (Figure 3B). However, *E. coli* were still distinguishable from the background signal through local maxima identification. When bacteria were enumerated in this way, the results matched the qualitative observation that *E. coli* sedimented at the bottom of the lumen (Figure 3A,B). Further information on the development of this peak-finding enumeration approach is provided in the Supplementary Information Section S7 (Figures S3–S7).

In utilizing an enumeration-based approach to track the *E. coli* population, maximizing the number of *E. coli* in focus was an important consideration. This was in part achieved by the low magnification, which resulted in a wide depth of field where colony-forming units were identifiable. However, as *E. coli* primarily resided on the lower luminal wall of the organoids with diameters of ~1–3 mm, colony-forming units spanned hundreds of microns in height and as such could not be captured in a single focal plane. To encompass the wide spatial distribution, two distinct focal planes were flattened into a single image, which was then used to count bacteria (Figure 3A,C). By identifying bacteria after flattening the image, bacteria discernable in both focal planes were not counted twice. Compared with a flattened z-stack of the entire lumen, using two images accounted for roughly 50% of the total identified bacteria.

Multiple focal planes were combined for initial and final measurements, as the location of the focal planes could be manually selected with brightfield imaging. During the perfusion, a single focal plane at a set height was imaged. Consequently, direct comparisons cannot be made between imaging during perfusion and pre-/post-perfusion imaging. This approach highlights the importance of consistent focal plane selection in large 3D environments given the sensitivity of bacteria quantification to z-height. Imaging more focal planes increases the consistency of luminal measurements. However, in high-throughput or high-frequency imaging applications, minimizing the selection of focal planes provides a quicker option that limits unnecessary light exposure.

### 3.3. GFP-Labeled E. coli-Perfused HIOs Experiment

**Figure 4:**
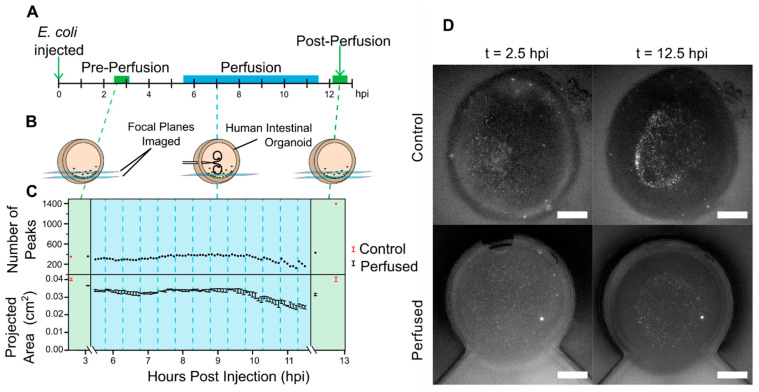
(**A**) A timeline for the perfusion experiment is shown. (**B**) Before and after the experiment, multiple focal planes were flattened to identify a maximal number of bacteria. During the perfusion period, only a single focal plane was used and kept at a constant height. The plotted data is for one HIO. (**C**) The number of peaks and projected area are shown with time. Single measurements are plotted as dots for number of peaks, and ±1 standard deviation for three area measurements is plotted for the projected area. Note: the first perfusion cycle is not included. Fluorescence imaging began 10 min after the first perfusion sequence. (**D**) Photos from before and after the experiment display settling for both the control and perfused HIOs. Scale bars: 500 μm.

This imaging strategy was applied to a 6.5-h perfusion of GFP-fluorescent *E. coli* (Figure 4A, Supplementary Video S2). During the perfusion, the *E. coli* remained primarily localized to the lower lumen and showed a relatively constant population. Bacteria counts did not show significant change with each perfusion cycle. Bacteria counts decreased near the end of the overall perfusion at 11 h post-injection (hpi), when the HIO had lost volume, thus shifting the relative location of the focal plane. Upon expansion to the original volume, the number of identified bacteria increased to slightly above the pre-perfusion count.

Before the perfusion experiment, control and perfused HIOs began with ~350 identifiable peaks. At the end of the perfusion experiment, the perfused *E. coli* population had grown to ~430 peaks, whereas the control population had grown to ~1400 peaks. On the premise that peaks accurately correlate with luminal bacteria concentration, the data indicate that there were fewer bacteria in the perfused HIO at 12 hpi.

The difference in bacteria count increase indicates that the perfusion system may have removed bacteria or limited bacterial proliferation through a different mechanism. Over the course of the perfusion, a volume roughly equivalent to nine times the luminal volume was exchanged within the HIO. Comparatively, the final segment of the dye perfusion with simultaneous, constant injection and withdrawal exchanged approximately four times the luminal volume. Despite the application of fluid flow in the perfused HIO, *E. coli* in both the control and perfused HIOs settled to the bottom of the lumen and aggregated in the central and lowest part of the lumen over time. This observation contrasts the luminal mixing observed with fluorescent dye, which diffuses rapidly and disperses homogenously, from the settling of larger particles that diffuse slowly.

Although further work is needed to investigate the mechanisms by which bacterial growth can be regulated, this experiment shows the potential for real-time imaging while interacting with and influencing a luminal environment. Bacterial enumeration is best suited for low bacterial concentrations where individual fluorescence intensity peaks can still be discerned. Physiologically, this situation would relate to bacterial concentrations found in the small intestines but not the large intestines [23,24]. Lower concentrations of bacteria are found in the small intestines due to the presence of gastric acid and its bactericidal ability, whereas the large intestines contain higher concentrations of bacteria to digest carbohydrates, proteins, lipids, and fiber that remain after processing by the small intestines.

### 3.4. Organoid Strain during Perfusion

During the perfusion experiments presented in this article, HIOs were observed to have small changes in size due to injection or withdrawal of liquid from the HIO lumen. Since the injection and withdrawal of media were sequential and not simultaneous, the injection of liquid caused a temporary increase in the diameter of the HIO and then the subsequent withdrawal caused the diameter to decrease from the enlarged state due to the injection. This resulted in cyclic expansion and relaxation of the HIO which was quantified by measuring the diameter of the HIO before injection, and subsequently measured again immediately after injection. The resulting change in diameter was used to calculate the cyclic tensile strain that the HIO cells experienced during the perfusion experiments. The average calculated strain during the experiments presented here was only ~1.7%, which was determined from the images of the three representative perfusion cycles of the experiment shown in Figure 2. The application of tensile strain to cell-based models has been previously used by others to better mimic living human intestinal function and has been shown to help promote accelerated intestinal cell differentiation, the formation of 3D villi-like structures, and increase the intestinal barrier function [25]. In these referenced intestine-on-chip devices, a 10% cyclic tensile strain was applied to the intestinal cells to simulate the peristaltic behavior of the human intestine. Although the strain applied due to perfusion in the experiments here was less than these previous intestine-on-chip devices, earlier versions of the perfusion system used in this paper demonstrated the perfusion system’s ability to create strains of ~9.3% on HIOs. Furthermore, the effect of perfusion with perfusion-induced cyclic strain can be used in the future to investigate other biological systems that can be grown as organoids and experience cyclic strain, such as lung [26] or bladder [27] models.

### 3.5. Histological Examination of Organoid Following Perfusion with E. coli

**Figure 5:**
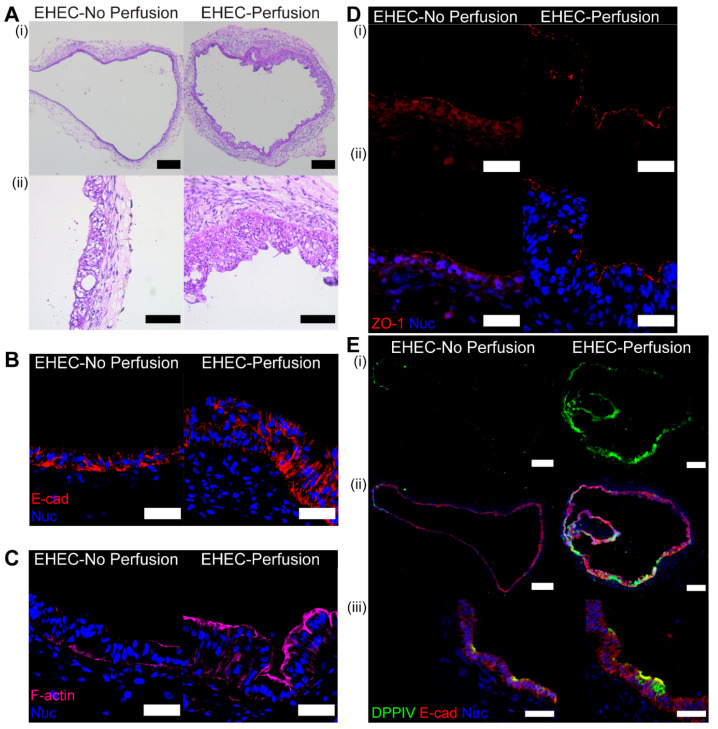
Immunostained cryosections of HIOs microinjected with enterohemorrhagic E. coli (EHEC) O157:H7. (**A**) H&E-stained sections of (left) unperfused HIOs and (right) perfused HIOs. Scale bars: (row **i**) 250 µm; (row **ii**) 100 µm. (**B**–**E**) Immunofluorescence-labeled cryosections of (left) unperfused HIOs and (right) perfused HIOs: (**B**) E-cadherin (red) and nuclei (blue); (**C**) F-actin (magenta) and nuclei (blue); (**D**) ZO-1 (red) and nuclei (blue). Row (**i**) shows only ZO-1 (red) and row (**ii**) overlays ZO-1 (red) and nuclei (blue); and (**E**) DPPIV (green), E-cadherin (red), and nuclei (blue). Scale bars: (**B**–**D**) 50 µm, (**E**, row **i**–**ii**) 250 µm, and (**E**, row **iii**) 50 µm.

After perfusion, HIOs were fixed and cryosectioned to observe responses to bacterial exposure and luminal fluid flow. Both H&E staining and E-cadherin (E-cad) staining (Figure 5A,B,E) showed the epithelial thickness of the perfused organoid was thicker than that of the control. The measured epithelial thickness of the perfused HIO was 53.2 ± 22.8 µm and the thickness of the unperfused control was 25.4 ± 10.3 µm (data presented in mean ± SD and measurement details shown in Supplementary Information Figure S7). Additionally, using a different perfusion sequence on a different HIO, similar epithelial thickening was observed. (Supplementary Information Figure S8).

*E. coli* O157:H7 is known to disrupt the intestinal epithelial barrier by remodeling actin filaments in the apical side and forming the locus of an enterocyte effacement (LEE) pathogenicity island. HIOs challenged with *E. coli* O157:H7 lose epithelial polarization within 18 hpi [28]. Similarly in our work, the epithelium of the *E. coli*-injected HIO without perfusion (static luminal environment) was losing structural integrity after 13 hpi (Figure 5C,D). When the *E. coli*-injected HIO was luminally perfused, however, the organoid was able to better retain its epithelial polarization (Figure 5C,D). Better polarization may be the result of a dynamic luminal fluid environment, which could have prevented the increase in the luminal bacterial concentration (Figure 4C). Additionally, the direct contact between *E. coli* O157:H7 and the apical surface of the epithelium may have been shorter with periodic convection. As the major virulence mechanism of *E. coli* O157:H7 is initiated by the attachment on the intestinal epithelial surface, reduced contact time may help delay the pathogenic effect of the bacteria.

In addition to structural integrity, the effect of perfusion on the functional maturation of the epithelium was also examined. Dipeptidyl peptidase 4 (DPPIV) is one of many enzymes expressed by enterocytes at the brush border to help digestion of peptides. Increased expression of DPPIV was observed in the perfused HIO compared with the control (Figure 5E). Commensal *E. coli*-injected HIOs without perfusion exhibited increased DPPIV expression at 48- and 96-hpi, whereas PBS-injected controls showed no detectable enzyme expression [18]. This could imply that luminal perfusion has a more immediate effect on DPPIV expression and the brush border maturation than stationary commensal colonization.

Gut motility is crucial for gastrointestinal health, as it not only helps digestion but also prevents bacterial overgrowth and helps pathogen clearance. In our study, luminal fluid flow changed how organoids responded to a pathogen both directly and indirectly. A limitation of this study is the lack of PBS-injected and perfused organoid control. However, previous studies using intestine-on-chip models showed apical fluid flow and stretching enhance the epithelial barrier function and stable colonization of probiotic or commensal bacteria [25], and infection of enteric pathogens [29]. Furthermore, previous studies have shown that microbes unable to proliferate in static in vitro intestinal models were able to successfully associate with human epithelium when appropriate mechanical cues were added before and after inoculation [29]. Collectively, our results and previous studies underscore the importance of peristalsis-mimetic stimulation in human–microbe interaction models.

## 4. Conclusions

Replicating the gastrointestinal environment requires integration of many physical, biochemical, and intra-organism interactions. To this end, perfusion devices enable new experimental manipulations to replicate physiologic conditions, such as applying strain to cells, removing cellular debris [9], and regulating luminal contents. Integrating perfusion devices into large 3D biological models such as organoids brings new technical challenges which this work addresses with key methodology choices. Our design utilizes a narrow and shallow-tapered double-barreled glass capillary to maximize success of puncture and perfusion. A pressure-based pump was opted for over displacement-based pumps to enable higher temporal control of luminal fluid injection and withdrawal. To image a large 3D volume, high-throughput bacterial enumeration methods that considered multiple focal planes were developed. Although this imaging method allows high throughput analysis, in general, injection-based perfusion systems require notable setup time. However, the utilization of double-barrel pulled capillaries in the presented perfusion system offers improvements to the throughput compared with systems utilizing two single-barrel capillaries [10] that require two separate alignment and piercing processes to provide perfusion of the lumen.

With these methodology choices implemented, our perfusion device shows potential for replicating gastrointestinal motility and microbiome stability. In perfusing an HIO with pathogenic *E. coli*, surprisingly large differences were observed for epithelial thickness, polarization, and DPPIV secretion. Further investigation is required to confirm biological changes observed in the HIO and isolate specific causes. Nevertheless, the observations lend support to the notion that the technology and methods described can have biological significance. Beyond HIOs, the perfusion technologies developed can be used to manipulate and investigate other biological models. In lung organoids, for example, perfusion may be used to model bacterial infection or apply physiologic strain. As more complex 3D in vitro models are developed, luminal perfusion technologies provide a promising approach for recapitulating physiologic conditions.

## Data Availability

Data is contained within the article; further inquiries can be directed to the corresponding authors.

## Supplementary Materials

The following are available online at https://www.mdpi.com/article/10.3390/mi13010131/s1, Supplementary Information, Figure S1: System diagram of pressure-based pump connected to perfusion device, Figure S2: (A) LabView used to analyze optical microscopy data of the droplet formation at the capillary tip. Flowrate of 3.78 µL/min obtained using this method. (B) Flow rate measurements obtained using time to fill a PDMS well method. Plot shows flow rate measurements of the capillary as function of applied pressure, Figure S3: Brightfield image of HIO colonized with *E. coli* in PDMS HIO holder overlayed with the peaks identified in the fluorescent image using the same peak finding algorithm employed in the HIO-*E. coli* coculture perfusion experiments in the main text. Group of large dark spots are dead cells within the lumen at the lower luminal wall, Figure S4: Bright field images of PDMS wells filled with *E. coli* and growth media. Images shown were captured at the top of the well, the bottom of the well, and at three intermediate heights (images are numbered in order from the top to the bottom of the well). Bacteria were identified using fluorescent images at each height and the same peak finding algorithm employed in the HIO-*E. coli* coculture perfusion experiments in the main text. The identified peaks (bacteria) are label by yellow plus symbols and overlayed on the images and the total peaks are listed for each image, Figure S5: Plot of the total fluorescent peaks identified at each of the five heights imaged in Figure S4. The number of peaks identified was the greatest near the bottom of the well, Figure S6: (left) Zoomed in image of region in PDMS well with E. coli and growth media shown in Figure S4 (Plane #5-Bottom). 73 peaks were identified manually within the oval region of interest. (right) Using the left image, 52 peaks were identified using the maxima finding algorithm utilized in the main text, Figure S7: Epithelial cell thicknesses measured and plotted of Figure 5E(ii) in the main text. Epithelial thickness of perfused HIO was 53.2 ± 22.8 µm and that of unperfused 25.4 ± 10.3 µm (data presented in mean ± SD). During measuring, 125 and 120 thickness measurements were performed for unperfused and perfused sections, respectively. Student’s t-test at α=0.05, *p* = 9.2 × 10^−28^, Figure S8: H & E stained sections of HIOs microinjected with EHEC: without intraluminal perfusion (left) and with perfusion (right). HIOs from different perfusion experiment than the main E. coli perfusion experiment in the main text and using a different perfusion sequence. Similar epithelial thickening observed. Scale bar: 100 µm, Table S1: Antibodies and reagents used for immunofluorescence staining, Video S1: *E. coli* perfusion, Video S2: FITC withdrawal.
