# Supplementary material for: Perfusion System for Modification of Luminal Contents of Human Intestinal Organoids and Realtime Imaging Analysis of Microbial Populations
Source: Micromachines (Basel). 2022 Jan 14;13(1):131. doi: 10.3390/mi13010131 (PMC8779378; doi:10.3390/mi13010131)
Supplement: Supplementary file 1 [file micromachines-13-00131-s001.zip › micromachines-1529372-supplementary-edited.pdf]

<u>**Supplementary Information**</u>

Perfusion System for Modification of Luminal Contents of Human Intestinal Organoids and Realtime Imaging Analysis of Microbial Populations

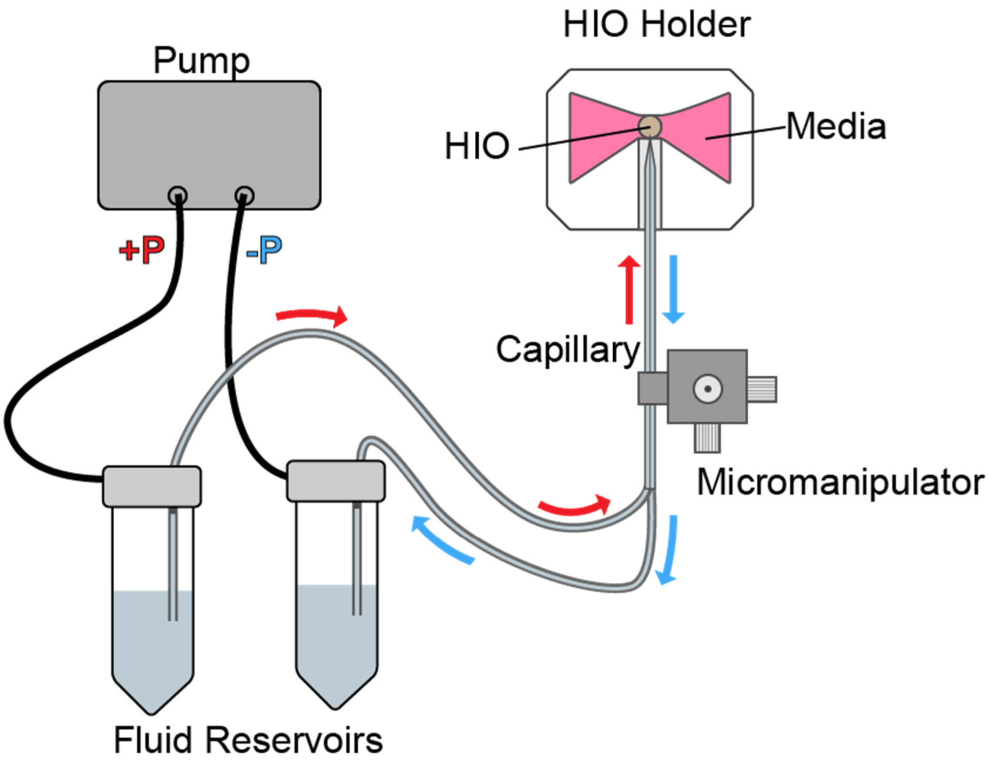

Figure S1. System diagram of pressure-based pump connected to perfusion device.

S1. Pipette Pulling

Pulling parameters were varied to find optimal pulling conditions; weights attached to the PC-10 Puller and heater settings were modified to produce the optimal tip geometry while maximizing the success rate of the procedure.

For perfusion experiments, double-barreled glass capillaries were pulled using a heater setting of 69.0 with 325 g attached weight. The resulting tip diameters were 120 µm and 105 µm for the FITC-dextran perfusion and the *E. coli* perfusion, respectively. For the *E. coli* injection, a single-barreled glass capillary was pulled at a heater setting of 60.0 with 250 g attached weight. The resulting tip diameter was 10 µm. Double-barreled glass capillaries formed a closure at the tip end, which was manually broken using a razor to achieve the desired tip end diameter.

S2. Capillary Tip Flow Rate Measurements

Flow rates of pulled glass capillary tips utilized by the perfusion system were calibrated and related to applied pressure using two different optical methods. The first method used visual inspection of the droplet formation at the capillary tip and fitting a circle to the time-series optical microscopy images to estimate the droplet volume over time. Using LabView (Figure S2A), best-fit circles were fit for every image frame, and droplet volume was approximated assuming a spherical shape of the droplet and subtracting the pipette volume contained within the droplet, as the droplet formed around the tip. A linear regression was performed upon this volume data. With +100 mbar applied using the Elveflow pump to a 110 µm total diameter capillary tip, the derivative of volume with respect to time was determined to be 3.78 µL/min with an R^2 value of 0.9998.

The second method to measure the flow rate was conducted by measuring the time to fill a known reservoir volume. This was performed by filling a Poly(Dimethyl Siloxane) (PDMS, Sylgard 184, Dow Corning) cylindrical well and optically verifying with a microscope when the well was filled or emptied. Pressures of +100, +200, -200, and -300 mbar were applied to a capillary with a 90 µm tip using the Elveflow pump, and the time to fill or empty the PDMS well was recorded for each pressure. A linear regression was fit to determine the corresponding flow rates (Figure S2B). From the four flow measurements at different pressures, the flow rate, Q, was linearly related to pressure, P, with the following relation: $Q = 0.0281 * P + 1.4979$ with an R^2 value of 0.9964. The intercept suggests that a positive flowrate is observed when zero pressure is applied, which likely resulted from a height differential between fluid wells and capillary tip.

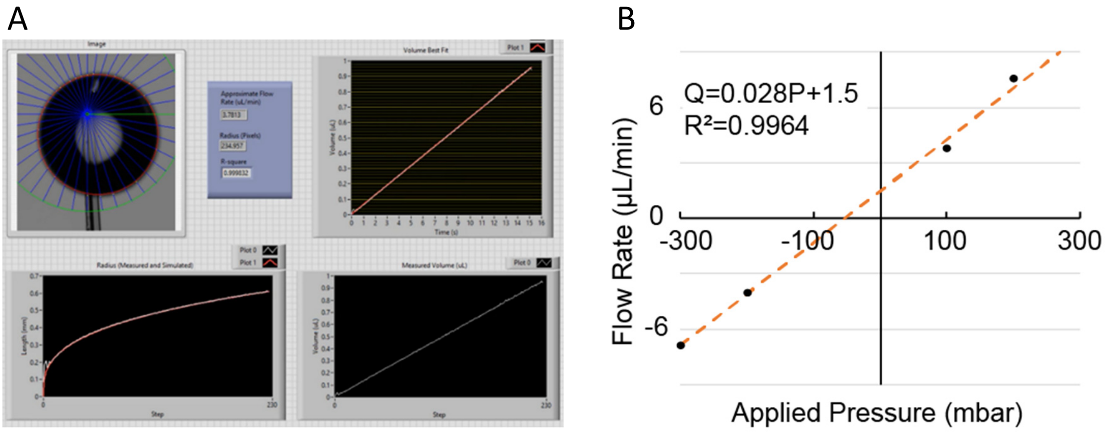

Figure S2. (A) LabView used to analyze optical microscopy data of the droplet formation at the capillary tip. Flowrate of 3.78 µL/min obtained using this method. **(B)** Flow rate measurements obtained using time to fill a PDMS well method. Plot shows flow rate measurements of the capillary as function of applied pressure.

S3. Dye Perfusion Sequence

A single perfusion cycle consisted of 3 seconds of +200 mbar applied to the injection barrel, followed by 5 seconds with no pressure, followed by 3 seconds of -200 mbar applied to the withdrawal

barrel. 20 cycles were performed in total, each separated by a period with no applied pressure averaging 1 minute, 37 seconds. Optical microscopy videos were recorded over the duration of each perfusion cycle, and fluorescent images were taken before and after each cycle. The first perfusion cycle was not imaged for fluorescence; brightfield video was recorded and inspected for any visual signs of leakage, which was not observed.

After 20 perfusion cycles were performed, the HIO had significantly expanded, so -200 mbar was applied to the withdrawal barrel for 15 seconds while no pressure was applied to the injection barrel. This was followed by -400 mbar applied for 40 seconds. Lastly, simultaneous injection and withdrawal (i.e. constant perfusion) was performed for 78 seconds by applying +200 mbar to the injection barrel and -400 mbar to the withdrawal barrel. For all additional pressures applied after the 20 perfusion cycles, fluorescent video was taken.

S4. *E. coli* Perfusion Sequence

To determine initial bacteria injection parameters with the MINJ-D microinjector (Tritech Research), droplets were created by applying positive pressure and were inspected under a stereomicroscope. Applying 130 kPa for 0.8 seconds created a droplet volume of ~0.065 μL, which was estimated with optical microscopy. This corresponded to an *E. coli* injection of ~650 CFU / HIO.

Unlike the dye perfusion experiment, a pressure of +25 mbar was required to produce zero net flow, as the fluid reservoirs were located below the height of the capillary and HIO. The pressure sequence applied to the capillary consisted of +200 mbar to the injection tip and +25 mbar to the withdrawal tip for 3 seconds, followed by +25 mbar applied to both tips for 5 seconds. Then +25 mbar was applied to the injection tip and -150 mbar was applied to the withdrawal tip for 3 seconds, followed by +25 mbar applied to both tips for 20 seconds. This injection and withdrawal sequence was repeated three times, after which +25 mbar was applied to both tips for a period of 28 minutes and 27 seconds. This entire 30-minute sequence was repeated for 6 hours 30 minutes, for a total of 13 perfusion cycles. Optical and fluorescent images were taken every 5 minutes. Similar to the dye perfusion, the first perfusion was not imaged, as brightfield video was inspected for signs of leakage, which was not observed. Following the 6-hour, 30-minute period, the HIO was expanded with positive pressure and optical and fluorescent images were taken.

S5. Microscope Imaging Settings

Perfusion imaging was performed on a DMi8 inverted microscope (Leica Microsystems). Fluorescent pictures and video were taken for the dye perfusion (excitation wavelength = 480/40 nm, emission wavelength = 527/30 nm, dichroic beamsplitter wavelength = 495 nm, exposure = 70 ms) and the *E. coli* perfusion (excitation wavelength = 470/40 nm, absorption wavelength = 525/50 nm, dichroic beamsplitter wavelength =495 nm, exposure = 70 ms.) The single-barrel *E. coli* injection was performed with optical guidance from a stereomicroscope (Leica Microsystems).

S6. Image Analysis Scripts and Plugins

S6.1. Brightness Calibration

```
import ij.IJ;
import histogram2.HistogramMatcher;
import ij.io.Opener
import ij.io.OpenDialog

// Use the OpenDialog to select a file.
filePath1 = new OpenDialog('Select an image file').getPath()
// Open the selected file.
imp1 = new Opener().openImage(filePath1)
// Display the ImagePlus.
imp1.show()

// Use the OpenDialog to select a file.
filePath2 = new OpenDialog('Select an image file').getPath()
// Open the selected file.
imp2 = new Opener().openImage(filePath2)
// Display the ImagePlus.
imp2.show()

ip1 = imp1.getProcessor();
ip2 = imp2.getProcessor();

hist1 = ip1.getHistogram();
hist2 = ip2.getHistogram();

matcher = new HistogramMatcher();
newHist = matcher.matchHistograms(hist1, hist2);

ip1.applyTable(newHist);
imp1.setProcessor(ip1);
```

S6.2. Stack Focuser Plugin:

Plugin found at https://imagej.nih.gov/ij/plugins/stack-focuser.html by Michael Umorin

S7. Bacteria Enumeration Investigations

S7.1. Determining Bacteria from Dead Cells

To provide support that fluorescence peaks are attributable to *E. coli* and not dead cells within the lumen, images of the perfused HIO colonized with *E. coli* were further analyzed to explore the possibility of dead cells contributing to maxima identification during image analysis. Figure S3 shows a brightfield image of the perfused HIO colonized with *E. coli* with dark spots on the lower luminal wall. It has been reported in previous studies that such dark spots in organoids are dead cells [1],[2]. The brightfield image of Figure S3 is overlayed with the peaks identified in the fluorescent image using the same peak finding algorithm employed in the HIO - *E. coli* coculture perfusion experiments in the main text. The image shows no obvious spatial correlation between the dead cells (dark spots) and the identified peaks and therefore supports the fluorescent peak finding process's ability to distinguish between bacteria and dead cells.

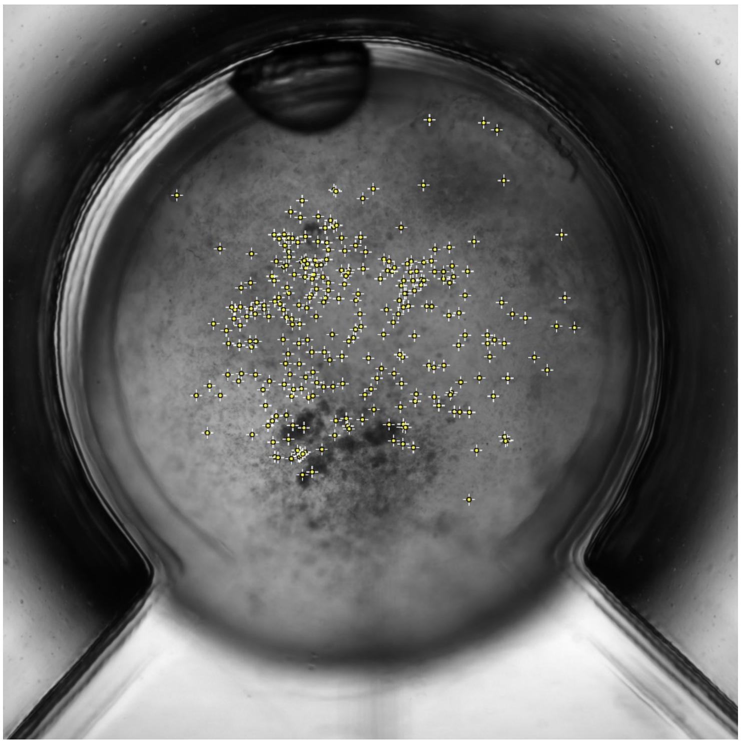

Figure S3. Brightfield image of HIO colonized with *E. coli* in PDMS HIO holder overlayed with the peaks identified in the fluorescent image using the same peak finding algorithm employed in the HIO - *E. coli* coculture perfusion experiments in the main text. Group of large dark spots are dead cells within the lumen at the lower luminal wall.

S7.2. Determining Bacteria from Media Autofluorescence

To provide support that fluorescence peaks are attributable to *E. coli* and not autofluorescence of the surrounding growth media, images of *E. coli* cultured in a PDMS well without an organoid were analyzed. The wells were 2 mm in diameter, 3 mm in height, and contained only bacteria and growth media. Fluorescent images were captured at the top of the well, the bottom of the well, and at three intermediate heights of the well. Bacteria were then identified using these images through the same peak finding algorithm employed in the HIO - *E. coli* coculture perfusion experiments in the main text. Figure S4 shows each of these five images at different heights in the well with the fluorescent peaks labeled along with the total number of peaks identified at each height. The images of Figure S4 and the plot in Figure S5 showing the total number of fluorescent peaks at each height, illustrate that the majority of bacteria identified are located at the bottom of the flat well. This agrees with the observation discussed in the main text that peaks were identified on the lower luminal wall of the HIO (Figure 3 of main text).

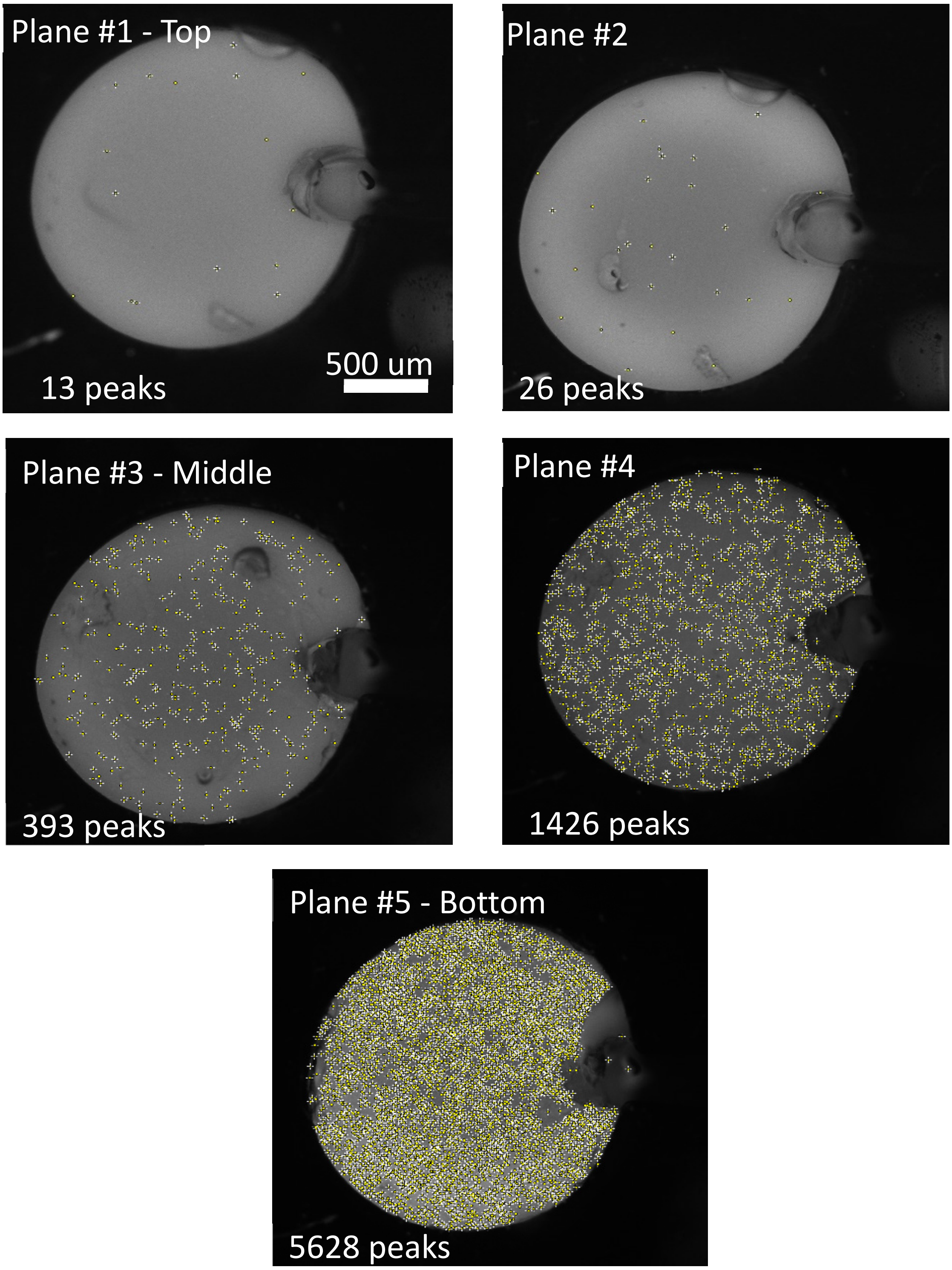

Figure S4. Bright field images of PDMS wells filled with *E. coli* and growth media. Images shown were captured at the top of the well, the bottom of the well, and at three intermediate heights (images are numbered in order from the top to the bottom of the well). Bacteria were identified using fluorescent images at each height and the same peak finding algorithm employed in the HIO - *E. coli* coculture perfusion experiments in the main text. The identified peaks (bacteria) are label by yellow plus symbols and overlayed on the images and the total peaks are listed for each image.

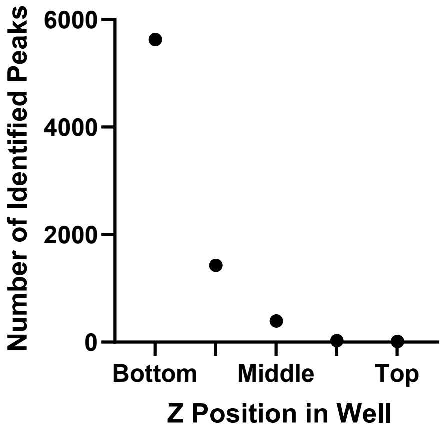

Figure S5. Plot of the total fluorescent peaks identified at each of the five heights imaged in Figure S4. The number of peaks identified was the greatest near the bottom of the well.

To provide additional confidence on the accuracy of the bacteria enumeration technique using fluorescent peaks, the peaks were analyzed in a specific region in the image at the bottom of the PDMS well of Figure S4. This zoomed in region of the well containing only *E. coli* and media is shown in Figure S6. Within the oval region in Figure S6, 73 peaks were identified manually using the left image of Figure S6, and the maxima finding algorithm identified 52 peaks in the same region (Figure S6, right image). This suggests that the 'prominence value' of 500 selected for this function provides a conservative estimate of bacteria within a region and can distinguish bacteria from the surrounding growth media's autofluorescence.

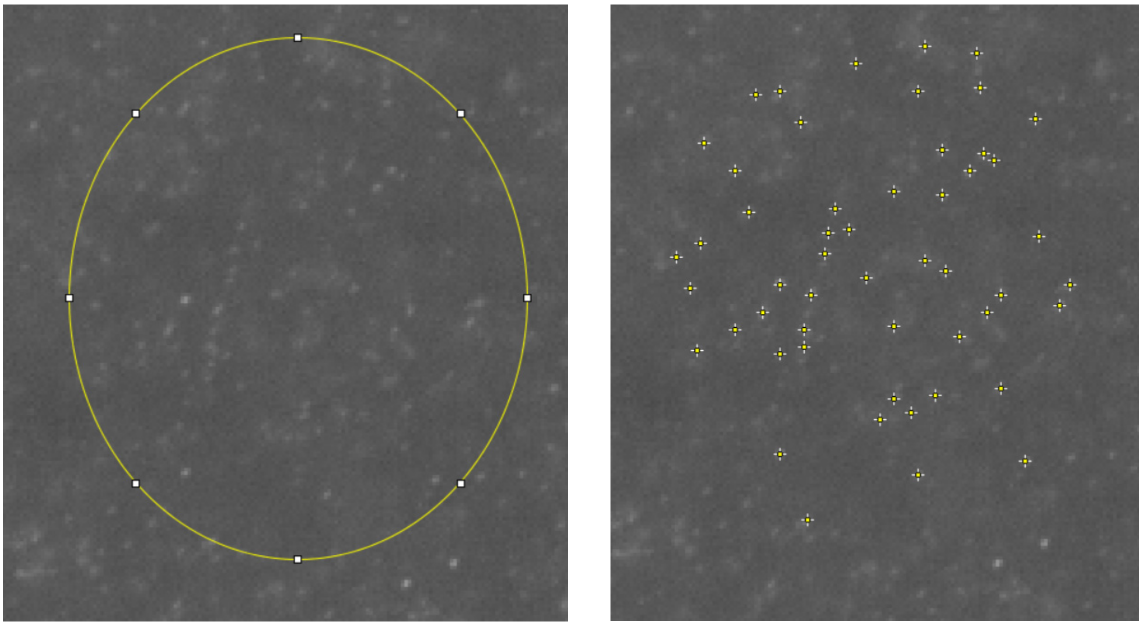

Figure S6. (left) Zoomed in image of region in PDMS well with *E. coli* and growth media shown in Figure S4 (Plane #5 – Bottom). 73 peaks were identified manually within the oval region of interest. (right) Using the left image, 52 peaks were identified using the maxima finding algorithm utilized in the main text.

Table S1. Antibodies and reagents used for immunofluorescence staining

Name	Supplier	Catalog Number	Dilution
Rabbit anti-E-cadherin	Cell Signaling	3195	1:200
Rabbit anti-ZO-1	Cell Signaling	13663S	1:200
Goat anti-Human DPPIV/CD26	R&D Systems	AF1180	1:100
Goat anti-Rabbit Alexa Fluor 594	Invitrogen	A-11012	1:1000
Donkey anti-Goat Alexa Fluor 488	Invitrogen	A-11055	1:1000
Alexa Fluor 594 Phalloidin	Invitrogen	A12381	1:40
DAPI	Invitrogen	D1306	1.43 µM
Bovine Serum Albumin (BSA)	Millipore Sigma	82-067	1-4% (w/v)
ProLong Diamond Antifade Mountant	Invitrogen	P36965	

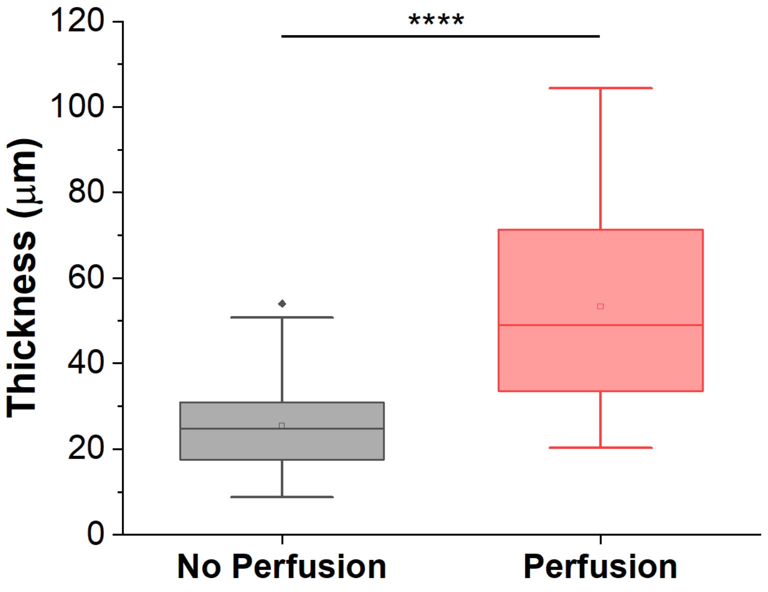

Figure S7. Epithelial cell thicknesses measured and plotted of Figure 5E(ii) in the main text. Epithelial thickness of perfused HIO was 53.2 ± 22.8 µm and that of unperfused 25.4 ± 10.3 µm (data presented in mean±SD). During measuring, 125 and 120 thickness measurements were performed for unperfused and perfused sections, respectively. Student's t-test at $\alpha=0.05$, $p=9.2E-28$.

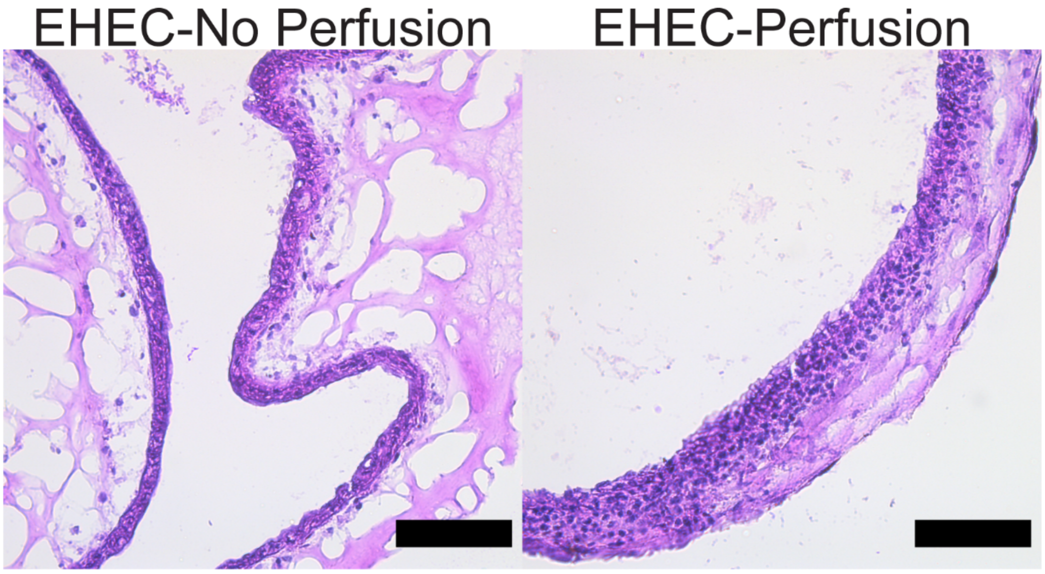

Figure S8. H&E stained sections of HIOs microinjected with EHEC: without intraluminal perfusion (left) and with perfusion (right). HIOs from different perfusion experiment than the main E. coli perfusion experiment in the main text and using a different perfusion sequence. Similar epithelial thickening observed. Scale bar: 100 µm.

References

[1] A. Andersson-Rolf, J. Fink, R. C. Mustata, and B. K. Koo, "A video protocol of retroviral infection in primary intestinal Organoid culture," *J. Vis. Exp.*, no. 90, pp. 1–8, 2014.

[2] A. Fatehullah, P. L. Appleton, and I. S. Näthke, "Cell and tissue polarity in the intestinal tract during tumourigenesis: Cells still know the right way up, but tissue organization is lost," *Philos. Trans. R. Soc. B Biol. Sci.*, vol. 368, no. 1629, 2013.
